# Endosymbiont diversity among sibling weevil species competing for the same resource

**DOI:** 10.1186/1471-2148-13-28

**Published:** 2013-02-04

**Authors:** Adrien Merville, Samuel Venner, Hélène Henri, Agnès Vallier, Frédéric Menu, Fabrice Vavre, Abdelaziz Heddi, Marie-Claude Bel-Venner

**Affiliations:** 1 Université de Lyon, Université Lyon 1, CNRS, UMR5558, Laboratoire de Biométrie et Biologie Evolutive, Villeurbanne, France; 2 INSA-Lyon, UMR203 BF2I, INRA, Biologie Fonctionnelle Insectes et Interactions, Bat. L. Pasteur 20 ave A. Einstein, Villeurbanne, France

**Keywords:** Endosymbiosis, Host community, Curculio, Oak weevil, Infection pattern, Niche partitioning, Field study

## Abstract

**Background:**

Whereas the impact of endosymbionts on the ecology of their hosts is well known in some insect species, the question of whether host communities are influenced by endosymbionts remains largely unanswered. Notably, the coexistence of host species competing with each other, which is expected to be stabilized by their ecological differences, could be facilitated by differences in their endosymbionts. Yet, the composition of endosymbiotic communities housed by natural communities of competing host species is still almost unknown. In this study, we started filling this gap by describing and comparing the bacterial endosymbiotic communities of four sibling weevil species (*Curculio spp*.) that compete with each other to lay eggs into oak acorns (*Quercus* spp.) and exhibit marked ecological differences.

**Results:**

All four species housed the primary endosymbiont *Candidatus* Curculioniphilus buchneri, yet each of these had a clearly distinct community of secondary endosymbionts, including *Rickettsia*, *Spiroplasma*, and two *Wolbachia* strains. Notably, three weevil species harbored their own predominant facultative endosymbiont and possessed the remaining symbionts at a residual infection level.

**Conclusions:**

The four competing species clearly harbor distinct endosymbiotic communities. We discuss how such endosymbiotic communities could spread and keep distinct in the four insect species, and how these symbionts might affect the organization and species richness of host communities.

## Background

The last decade has seen a growing number of studies exploring microbial endosymbiosis among arthropods and its implication for the evolution of the host species. The impressive array and diversity of species harboring one or more type(s) of endosymbionts raises the possibility that such associations may play a predominant role in the ecology and evolution of both partners. Most studies that have considered symbiotic associations from the perspective of the host support the view that heritable symbionts offer their host an opportunity for rapid and deep evolutionary changes and for greater adaptation to a novel environment [1-3]. Symbionts are considered to be a keystone in the diversification and expansion of the ecological niche of their host [3-5]. Thus, endosymbionts have been shown to assist their host in exploiting poor nutritional resources, notably by complementing its nutrition [6-8], often to the point that they become essential or obligatory symbionts for the host. From the differences observed between the endosymbiotic communities of various host populations, it has been suggested that symbionts may mediate the specialization of their hosts on distinct resources and thus promote the emergence of allopatric host races, which might subsequently lead to speciation [9-15].

Mutualistic facultative symbionts are also able to provide their host with other skills, such as broadening the range of temperatures they can tolerate [16], reinforcing their ability to resist natural enemies [17-20], or increasing their dispersal capacity [21-24]. These rapidly-acquired capacities are likely to promote the expansion of the host populations and the diversification of their habitat [1-5]. Besides these mutualistic relationships, facultative endosymbionts are also known to manipulate the reproduction of the host. In response to selective pressures favoring their vertical transmission, many symbionts, such as *Wolbachia*, have been shown either to skew the sex-ratio of the offspring toward females or to induce sterilization of uninfected females [25-27]. Such effects can affect both the population dynamics and gene flow, notably by promoting the reproductive isolation of infected hosts.

While firm knowledge is accumulating about the symbiont-mediated interactions between given host species and their environments, the possibility that endosymbionts may influence the organization of communities composed of several competing host species has so far been seldom investigated. The first empirical evidence of an endosymbiont-mediated relationship between competing host species has been recently provided from artificial protist communities kept under highly-controlled laboratory conditions. It was suggested that a green algal symbiont is essential for competing protist species to coexist, notably because the allelochemicals and the nutritional resource provided by this microorganism seem to balance the competitive capacity of the hosts [28]. Moreover, the issue of the competition between two hymenopteran parasitoid species has been shown experimentally to be tightly dependent on the presence of an inherited virus in one of the insect species [29]. These findings highlight the fact that the influence of microbial symbionts on the organization of competing host communities may have been considerably underestimated. The paucity of empirical data about the role of endosymbionts on competing host communities contrasts with the body of knowledge that has accumulated about extracellular symbiosis that has been shown to play a key role in the organization and composition of plant communities [30-32]. Microbial communities present in the soil often mediate nutrient uptake of plants, and might facilitate the coexistence of plant species in various ways, notably by allowing resource partitioning (reviewed in [33]).

A first support to the hypothesis that endosymbiosis does impact communities of insect species competing with each other would be that endosymbiotic profiles vary with respect to the host species. To study this aspect, we investigated endosymbiosis in natural oak weevil communities composed of four *Curculio* species – *C*. *glandium* (Marsham), *C*. *elephas* (Gyllenhal), *C*. *pellitus* (Boheman), and *C*. *venosus* (Gravenhorst) (Coleoptera, Curculionidae). These weevils coexist on the same individual oak trees (*Quercus* spp.) in southern Europe [34-36], where they compete for oak acorns that constitute the sole food resource for larval development and that have been shown to be highly limiting some years [37]. This biological system is relevant for examining our proposal in that (*i*) the four oak weevil species are expected to host endosymbionts, like the few other *Curculio* species already described [15,38] and (*ii*) they exhibit marked differences in their life history traits despite their recent divergence [36], suggesting that their coexistence is stabilized by means of ecological niche partitioning [37].

We provide an exhaustive description of the endosymbiotic meta-community harbored by the four oak weevil species in two distinct natural communities. Using a correlative and a quantitative approach, we found that endosymbiont communities differed across the four *Curculio* species, while being consistent within each species. We discuss the mechanisms possibly accounting for the contrasted infection patterns observed across these sibling species and the ways by which endosymbiosis may contribute to structuring communities of insect species competing with each other.

## Methods

### Study system

Four weevil species of the genus *Curculio* (*C*. *glandium*, *C*. *elephas*, *C*. *pellitus* and *C*. *venosus*) coexist on oak trees (*Quercus* spp.) in southern Europe [34]. In all four species, females lay eggs during summer within oak acorns, where larvae achieve their development before self-extracting and burrowing into the soil. Fully mature larvae then enter diapause during variable time periods -*i*.*e*., they spend from one to four years underground-, depending on the species or even on individuals within one species [37].

### Insect sampling

Our study was conducted on two natural communities found in two sites in southern France (site 1: N45° 35’; E5° 01’; site 2: N45° 45’; E5° 16’). Adult weevils were live-trapped on oak trees at each site weekly throughout one breeding season (June to September 2009) by beating branches with a wooden stick [39]. Insects were collected on a white sheet laid under the tree and their species was identified from morphological traits [34].

### DNA extraction

Ovaries of 391 adult females caught on one of the two study sites and belonging to one of four weevil species (see Table 1) were dissected in buffer A (KCL 25 mM, MgCl2 10 mM, Saccharose 250 mM, Tris–HCl 35 mM; pH = 7.5) and homogenized in 500μL of buffer STE (NaCl 100 mM, Tris–HCl 10 mM and EDTA 1 mM, pH = 8). These dissected tissue samples were individually subjected to DNA extraction by adding proteinase K (120 mg/L) and SDS (sodium dodecyl sulfate: 0.5% m/v), and were then incubated for 2 h at 55°C. RNA contamination was removed with the addition of RNaseA (60 mg/μL) for 1 h at 37°C. DNA from each sample was then purified with phenol-chloroform extraction and precipitated using isoamelic alcohol [40]. In addition, 171 adult males (Table 1) were automatically crushed individually with stainless steel beads shaken 20s at 20Hz (Tissue Lyser, Qiagen) [41] and their total DNA was extracted using NucleoSpin Tissue Kit (Macherey-Nagel).

**Table 1:** Number of adult males and females of each weevil species collected at the two sites

**Species**	**Females**		**Males**	
	**Site 1**	**Site 2**	**Site 1**	**Site 2**
*C. elephas*	74	38	25	21
*C. glandium*	76	40	26	29
*C. pellitus*	78	7	36	7
*C. venosus*	64	14	15	12

### Characterization of the endosymbiotic lineages and phylogenetic analyses

To get an overview of the endosymbionts harbored by the four *Curculio* species, bacterial *16S rRNA* amplification was performed individually on the ovaries of 95 females among which 40 *C*. *elephas* (24 from Site 1 and 16 from Site 2) and 20 females of each of the three remaining species (*C*. *glandium*, *C*. *pellitus* and *C*. *venosus*) comprising 10 females per site, except for *C*. *pellitus* from the site 2 for which only 5 females could be sampled. A 1.5 kb fragment was amplified using eubacterial universal primers (Table 2). Reactions were carried out in a 50μL final volume consisting of 2.5 units of Taq DNA polymerase (UptiTherm, Interchim, France), 2 mM MgCl2, 0.8 mM dNTPs, 0.6 μM primers, and 200 ng DNA template. The polymerase chain reaction (PCR) started at 95°C for 5 min and was followed by 26 cycles, each of these running 95°C for 30s, 53°C for 1 min and 72°C for 2 min; the final step of the reaction was 7 min at 72°C. The PCR products were then pooled into eight groups, one per species and per site. Each of these pools was then subjected to cloning. In a first step, ten clones per pool were randomly selected and sequenced (Additional file 1; Beckman Coulter Genomics, Grenoble, France). Identifying these sequences revealed a predominance of three endosymbiont types (among which, mostly *Curculioniphilus buchneri*, and to a lesser extent, *Wolbachia* and *Rickettsia*; see below). Consequently, in a second step at least 130 clones were grown up for each pool (Additional file 1), and each clone was screened by diagnostic PCR using the specific primers corresponding to the three dominant bacterial types (Table 2). Clones that were grown successfully but that could be amplified by none of these three diagnostic PCRs (*i*.*e*., *Curculioniphilus buchneri*, *Wolbachia* and *Rickettsia*) were then sequenced (30 clones; see Additional file 1).

**Table 2:** Primers used for PCR diagnostic

**Target Species**	**Target gene**	**Primer name**	**Primer sequence (5’-3’)**	**Product size (kb)**	Temp.^*^(°C)	**Refs.**^**†**^
*Host*	CytB rRNA	CBJ-10933	TATGTACTACCATGAGGACAAATATC	0.5	45	[42]
*(Curculio sp.)*		CBN-11367	ATTACACCTCCTAATTTATTAGGAAT			[42]
*Eubacterial Universal Primers*	16S rRNA	008For	AGA GTT TGA TCA TGG CTC AG	1.5	53	[43]
		1487Rev	TAC CTT GTT ACG ACT TCA CC			(**)
*Candidatus* Curculioniphilus buchneri	16S rRNA	16S-F	AGAGATCTGGAGGAATATCA	0.4	52	(*)
		16S-R	CACTAAAGCATCTCTGCTAAAT			(*)
	*GroEL*	GroEL 2 F	ATG GGB GCT CAA ATG GTK AAA	0.9	55	[38]
		GroEL 2R	CTCTTTCATTTCAACTTCNGTBGCA			[38]
*Wolbachia*	16S rRNA	W-Spec F	CATACCTATTCGAAGGGATAG	0.4	60	[44]
		W-Spec R	AGCTTCGAGTGAAACCAATTC			[44]
*Rickettsia*	16S rRNA	RbF	GCTCAGAACGAACGCTATC	0.9	58	[12]
		RbR	GAAGGAAAGCATCTCTGC			[12]
*Spiroplasma*	ITS and 16S rRNA	Spixo-16S F	TTAGGGGCTCAACCCCTAACC	0.8	52	[45]
		Spixo-16S R	TCTGGCATTGCCAACTCTC			[45]
*Sodalis*	16S rRNA	Sodalis 370 F	CGRTRGCGTTAAYAGCGC	0.2	55	[38]
		16SSod590R	AACAGACCGCCTGCGTACG			[38]

*Annealing temperature. ^†^References: (*) this study; (**) nucleotides numbering from Escherichia coli, GenBank accession number J01859.

We characterized the bacteria hosted by *Curculio* females by performing phylogenetic analyses using the sequences obtained in this study together with the most similar ones found by Blast in Genbank. Multiple sequences were aligned using MUSCLE software [46]. The appropriate model of evolution was estimated with jmodeltest [47] for each set of sequences considered. Phylogenetic analyses were performed using maximum likelihood (ML) inference with Phyml v3.0.1 [48]. The models selected were *GTR* + *G* for *Rickettsia* (*16S*, 873 sites) as well as for *Spiroplasma* (*16S* + *ITS*, 1405 sites), *HKY85 + I* for *Serratia* (*16S,* 503 sites), *K80 + I f*or *Wolbachia* (*16S*, 340 sites)*.*The robustness of the nodes was assessed with 100 bootstrap replicates. Additionally, Bayesian phylogenies were performed with MrBayes v3.1.2 [49] using appropriate parameters leading to convergence between two runs. We then compared topologies obtained with the two methods using approximate unbiased (AU) [50] and Shimodaira and Hasegawa (SH) [51] tests available in the CONSEL program package [52]. No significant difference was detected between the two topologies for any of the bacteria.

To examine the possible co-diversification between *Curculioniphilus buchneri*, considered as the primary symbiont, and its hosts, we used the method previously described to build the phylogenies based on *COI* gene sequences retrieved from Genbank for the weevil phylogeny, and on two distinct markers, the *16S rRNA* sequence and the *GroEL* gene, for the bacterial phylogeny. Nine host-symbiont pairs were included in the analysis, among which the four *Curculio* species studied here, the hazelnut weevil *C. nucum* that we collected in a French population near Lyon, and four Japanese Curculio species whose primary symbiont has already been described (*C. cameliae*, *C. sikkimensis*, *C. robustus* and *C. dentipes*; [38].). The models selected were *TPM2 + I* for *C. buchneri* (*16S,* 267 sites), *GTR + I + G* for *Curculioniphilus buchneri* (*GroEL* gene, 915 sites) and for *Curculio* spp*.* (*COI* gene, 375 sites). For *Curculioniphilus buchneri* bacteria, the congruence of the *16S rRNA* and the *GroEL* topologies was rejected by the reciprocal AU and SH tests (AU: *P* = 0.004; SH: *P* = 0.014) probably because the *16S* topology was weakly resolved, which precluded the concatenation of both sequences. Then, we tested the global congruence between the symbiont and host phylogenies, using *16S* and *GroEL* gene for the bacterial tree separately, with the CopyCat program (The Cophylogenetic Analysis Tool, version 2.00.02 [53]). After checking the occurrence of global co-diversification, we further tested the association of each of the nine individual host-symbiont pairs using the *COI* and the *GroEL* phylogeny that provided the best resolved bacterial tree. Patristic distances were used as input matrix and the AxParafit program was run with 999 permutations [54].

### Diagnostic PCR

After checking the quality of the DNA of each insect sample (by amplifying a 487 bp fragment of the *cytochrome b* gene of the insect; Table 2), 562 adult weevils (males or females from one of the four species; see Table 1) were screened for each of the five endosymbionts found in the first part of the study using their specific primers (*i.e.*, *C. buchneri*, *Wolbachia*, *Rickettsia*, *Spiroplasma* and *Sodalis*; Table 2)*.* Although diagnostic PCR might fail to detect an endosymbiont that might be too rare in the host individual, we considered in subsequent analyses that the insect was free of this bacterium. To check whether the successful PCRs were actually specific of the targeted symbiont, two DNA amplicons randomly selected per symbiont and per weevil species were sequenced. To discriminate between the two possible *Wolbachia* strains detected in our samples (see Results), we performed a Restriction Fragment Length Polymorphism following the PCR assay based on the *16 s rRNA* sequence (PCR-RFLP) on each *Wolbachia*-infected weevil. For that purpose, 17 μl of each PCR product was digested at 37°C for 3 h with 5U of *AluI* (Fermentas, Villebons/Y., France) and was separated on a 2% agarose gel electrophoresis during 1 h at 100 V. One DNA sample from each *Wolbachia* strain and each host species was randomly selected and sequenced to check the accuracy of the bacterial lineage identified by PCR-RFLP.

### Data analysis

In each weevil species, we used a generalized linear model (GLM) fitted with a binomial error and a logit-link function to test whether the probability for an insect to be infected by the predominant facultative endosymbiont depended on its sex, on the locality it came from, or both. The best-fitted model was selected for each endosymbiont according to the AIC criterion. Further, the effects of each variable selected in the model and the possible interactions between variables were tested by an analysis of deviance. All the analyses were performed with the R software v.2.12.0 (http://www.r-project.org/).

## Results

### Endosymbiotic communities in the oak weevil communities

**Figure 1:**
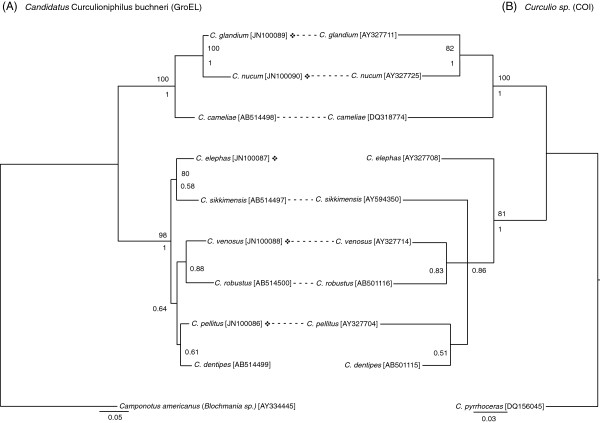
**Phylogenies of the *Curculio *species and their primary symbiont.** The phylogeny of *Curculioniphilus* is based on the *GroEL* gene, as its topology was better resolved than that obtained with the *16S rRNA* sequence. The *Curculio* phylogeny is based on a 375 bp sequence of the *COI* gene. The Bayesian trees are shown, as the Maximum-likelihood trees (not shown) exhibit substantially the same topology (see Methods). The bootstrap values for the maximum-likelihood analysis (100 replicates) and the Bayesian posterior probabilities are shown above and below the nodes only if greater than 50 and 0.50, respectively. The accession numbers of the nucleotide sequences are shown in brackets. Sequences indicated with asterisks were obtained in this study, the other sequences being retrieved from Genbank. The name of each bacterial sequence corresponds to that of its host. The association between the host-symbiont pairs is indicated by dotted line only when significant (*i.e.*, all significant associations were detected with less than a 0.02 risk error except for *C. sikkimensis* (0.026) and *C. venosus* (0.034) and their symbionts; see text for the method).

**Figure 2:**
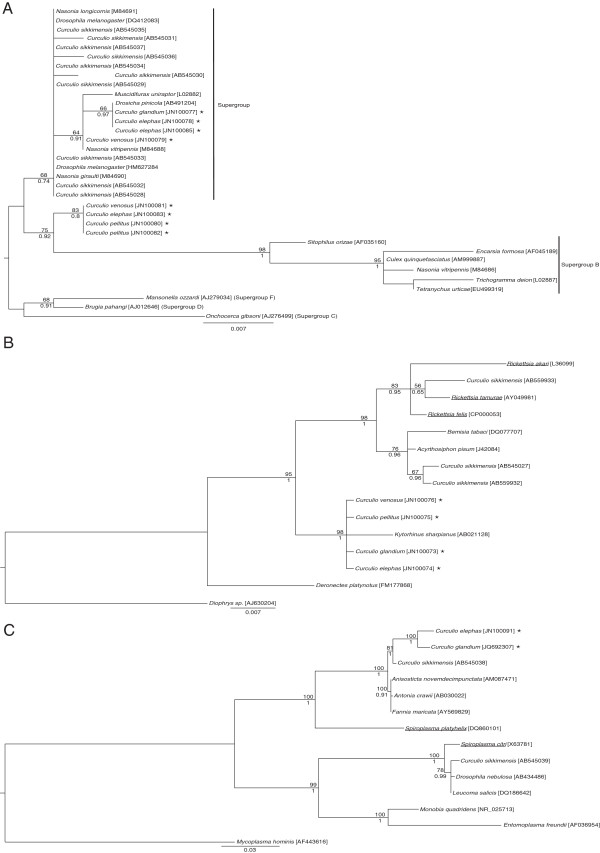
**Phylogenies of the facultative endosymbionts identified in *Curculio *spp.** (**A**) *Wolbachia* (340 bp unambiguously-aligned nucleotide sites, *16S rRNA* sequences) is shown with indication of the supergroups A and B based on the annotation of sequences found in Genbank; (**B**) *Rickettsia* (873 sites, *16S rRNA* sequences); (**C**) *Spiroplasma* (1405 sites, *ITS* and *16S rRNA* sequences). The Bayesian trees are shown, as the Maximum-likelihood trees (not shown) exhibit substantially the same topology (see Methods). The bootstrap values for the maximum-likelihood analysis (100 replicates) and the Bayesian posterior probabilities are shown above and below the nodes only if greater than 50 and 0.50, respectively. The accession numbers of the nucleotide sequences are shown in brackets. Sequences obtained from this study are indicated by asterisks. The name of each bacterial sequence corresponds to that of its host. Bacteria reported as non-endosymbiotic ones in the literature are underlined.

On the basis of 1,358 *16S rRNA* clones derived from the ovaries of females of the four oak weevil species collected at the two study sites, ten distinct bacterial lineages were identified (Additional file 1): *Curculioniphilus buchneri*, *Wolbachia, Rickettsia*, *Spiroplasma, Sodalis, Serratia, Xenorhabdus, Brevundimonas, Erwinia* and *Escherichia*. *Xenorhabdus, Brevundimonas* and *Erwinia* are known to be entomopathogenic and/or free-living bacteria. The identified strain of *Escherichia* corresponds to the vector used for the cloning step. The phylogenetic analysis of *Serratia* reveals that it is part of a monophyletic group of non-symbiotic bacteria that are distant from those known as symbionts (data not shown). Furthermore, this bacterium was shown to infect very few insect individuals at any site and irrespective of the weevil species, suggesting that the *Serratia* strain identified in this study was likely to be an insect pathogen: we discarded it from further analysis. A single clone among 1,358 ones was identified as a *Sodalis*-like endosymbiont (97,77% of similarity with the *Sodalis* strain found in *C. sikkimensis*). It was detected in the pool of *C. elephas* females sampled at the site 2, and could be *a posteriori* detected by means of individual PCR diagnostic in one of the 16 females of this pool. We could not detect this symbiont in any of the 562 oak weevils individually screened with the same method. Consequently, we conducted phylogenetic analyses on the five endosymbionts identified (i.e. *Curculioniphilus*, *Wolbachia* strains 1 and 2*, Rickettsia*, *Spiroplasma*) (Figures 1 and 2).

For the *Curculioniphilus* endosymbiont, four distinct sequences were obtained, one for each weevil species. We found statistical evidence of global co-diversification between weevil hosts and their primary symbiont on each dataset (*P* = 0.002 for *COI*-*GroEL* comparison, *P* = 0.005 for *COI*-*16S* comparison). In addition, the association of seven of the nine host-symbiont pairs individually tested was found significant (see Figure 1). The insignificant associations corresponded to *Curculio elephas* and *C. dentipes* with their symbionts, and could be explained by their weakly supported positions in the phylogenetic trees generated either by bootstrap or by Bayesian posterior probabilities. This finding suggests co-diversification between *Curculioniphilus* and its weevil hosts and implies that this endosymbiont has been acquired by a common ancestor of *Curculio* spp. and subsequently strictly vertically transmitted. The *Wolbachia 16 s rRNA* sequences revealed at least two distinct strains. The corresponding phylogeny (Figure 2A) indicates that one of them belongs to the *Wolbachia* supergroup A while the other one seems rather positioned at the edge of the *Wolbachia* supergroup B, according to the way *Wolbachia* sequences are annotated in Genbank. While we detected *Rickettsia* in all four weevil species, we failed to discriminate them from one host species to the next. The phylogenetic analysis (Figure 2B) suggests that this bacterium is closely related to the *Kytorhinus sharpianus* endosymbiont (Coleoptera: Bruchidae, [55]). Finally, 3.33% and less than 1% of the bacterial clones derived from *C. elephas* and *C. glandium* females contained *Spiroplasma*, respectively: this bacterium was allied to the *Curculio sikkimensis* endosymbiont (Figure 2C, [15]).

### Individual and multiple infection status of the oak weevils

**Figure 3:**
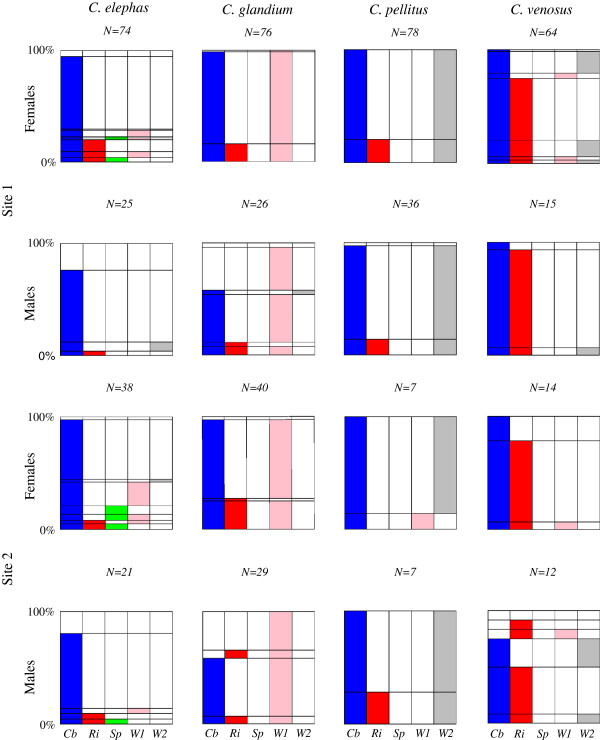
**Individual multiple infection status observed in two French communities of acorn weevils.** Five different endosymbionts have been detected in the four *Curculio* species studied: (Cb) Blue - *Candidatus* Curculioniphilus buchneri, (Ri) Red -*Rickettsia*, (Sp) Green-*Spiroplasma*, (WA) Pink - *Wolbachia* strain A and (WB) Gray - *Wolbachia* strain B. The geographical origin and the sex of the weevils are indicated on the left of the figure. The number of individuals tested is shown at the top of each graph. Horizontal reading informs us about the proportion of individuals sharing a given infection status. The prevalence of a given bacteria in a sample (of a given sex, site, species) is obtained by summing vertically the corresponding color.

We have screened *Curculioniphilus* as well as the three other endosymbionts in each of 562 weevils collected (Table 1). *Curculioniphilus* is highly prevalent in adults from the four species irrespective of the sex or the study site (it was found in 90.5% *C. elephas,* 84.2% *C. glandium,* 98.4% *C. pellitus and* 97.1% *C. venosus*; see Figure 3). The few uninfected individuals were essentially males (37 out of 44 uninfected individuals). Besides this so-called primary symbiont, three weevil species were shown to house their own predominant facultative symbiont (see Figure 3): 97.1% of 171 *C. glandium* individuals were infected by the *Wolbachia* strain belonging to the supergroup A, 98.5% of 128 *C. pellitus* individuals were infected by the *Wolbachia* strain at the basis of the supergroup B, and 77.1% of 105 *C. venosus* individuals hosted *Rickettsia*. In none of these species did the prevalence of the predominant secondary symbiont significantly vary according to either the sex or the study site (Table 3). A significant effect of the sex:locality interaction was statistically detected, however, on the prevalence of the supergroup A *Wolbachia* strain in *C. glandium*. This effect seems to be due to the unequal infection rate observed mostly in males between the two sites (infected males: 87% (site 1, n = 26) *vs* 100% (site 2, n = 29); infected females: 100% (site 1, n = 76) *vs* 95% (site 2, n = 40); Figure 3). This finding might stem from an unbalanced distribution of aged males in the two sites sampled (see discussion), and seems to be of low relevance owing to the small sample size of males as compared with that of females. In the two oak weevil communities studied, *C. elephas* differed from the three other species in that it lacked a predominant symbiont and exhibited *Spiroplasma* in addition to the three other secondary symbionts, all four endosymbionts being detected at a residual frequency. The three other weevil species, in addition to their predominant secondary symbiont, also harbored the other endosymbionts -except *Spiroplasma*- at a residual frequency. Considering the individual host level, multiple infections commonly occurred in the four species, mostly due to the co-occurrence of the primary symbiont and the predominant secondary symbiont (Figure 3). Interestingly, the two strains of *Wolbachia* were never detected simultaneously in any individual despite their marginal co-occurrence within the same species and study site.

**Table 3:** Probability for a predominant facultative symbiont to infect an individual host as a function of its sex and of the study site

**Variable**	**Deviance**	**df**	**P**
*Rickettsia* (*C. venosus*)			
Site	0.31589	1	0.57409
Sex	0.66578	1	0.41453
Site:Sex	2.75129	1	0.09718
*Wolbachia* A (*C. glandium*)			
Site	0.0003	1	0.98704
Sex	1.746	1	0.18635
Site:Sex	8.9503	1	**0.00277**
*Wolbachia* B (*C.pellitus*)			
Site	1.9356	1	0.16415
Sex	0.0747	1	0.78467
Site:Sex	3.7132	1	0.05398

Each analysis was performed on the weevil species shown in brackets. For each bacterium, the best-fitted generalized linear model (logistic regression) is shown, and was selected on the basis of the Akaike Information Criterion (AIC =117.2 for *Rickettsia*, 42.48 for *Wolbachia* A and 22.88 for *Wolbachia* B).The P-values for respective explanatory variables were obtained with chi-square statistics.

## Discussion

This study thoroughly describes the endosymbiotic communities hosted simultaneously by four weevil sibling species that coexist, compete with each other for a limiting resource (*i.e*., oak acorns as egg-laying sites) [37,56,57] and display contrasted ecological traits [37,58]. We found that while all four species hosted the same primary endosymbiont, they harbored markedly distinct communities of secondary endosymbionts. Even if our results are strictly correlative, since we did not investigate the actual role of each symbiont on its host, this finding is compatible with our proposal that stable coexistence of host species competing with each other, which is expected to be ensured by their ecological differences, might be mediated by their endosymbionts.

*Curculioniphilus buchneri* was identified in all four weevil species using an *a priori*-free method based on *16S rRNA* cloning and sequencing. This symbiont is known to be part of a distinct clade of Gammaproteobacteria, and is hosted by as many as 9 *Curculio* species. These insects feed on achenes or capsules and are specialist of perennial plants, including Theaceae (*Camellia* spp.) and Fagaceae (*Corylus*, *Castanea*.and *Quercus* spp.) [36,38]. The congruence observed between the phylogenies of *C. buchneri* and that of their host (Figure 1) suggests that this symbiont has been ancestrally acquired [4], which sustains its primary status. *C. buchneri* was found at a very high infection rate in the weevils screened in this study, and the few insects that did not carry *C. buchneri* were almost exclusively males (Figure 3). As primary endosymbionts are most often involved in complementing the host’s diet, this finding suggests that this bacterium might have been lost during adulthood by a few males and hence, that it might be essential to the larval rather than the adult stage [59].

Three of the secondary symbionts (two distinct clades of *Wolbachia* and one *Rickettsia*) were commonly detected in all four species, whereas the fourth one, *Spiroplasma*, was found exclusively in *C. elephas*. This pattern resembles that of an endosymbiotic metacommunity, in which the endosymbiotic communities of each host population/species comprise all the bacterial types and are connected to one another by means of horizontal transfers [2,4,60-65]. Horizontal transfers have been reported to occur between parasitoid species sharing the same host [65], and are likely to occur between the four weevil species under study here, because of the concomitant presence of larvae from several species occasionally feeding on the same individual acorns (unpublished results). Evidence of such transfers remains to be established, however, notably through the analysis of more variable molecular sequences of the symbionts.

While at first sight the set of secondary endosymbionts detected was found to be identical across the four weevil species, a finer-tuned, quantitative analysis revealed that these ones had clearly distinct endosymbiotic communities. The four insect species differed from one another in that, with the exception of *C. elephas,* each had its own major secondary symbiont. Two questions remain to be addressed: (i) why not all symbionts have spread to all four weevil species, and (ii) why different symbionts predominate in the different host species? Multiple infections are likely to be counter-selected since at the individual host level, competition between endosymbionts for the resource, might lead to competitive exclusion and/or to increased cost for the host [66,67]. Although we observed the presence of a particular endosymbiont correlating with a weevil species, we cannot infer at this time its actual impact on host phenotype. However, assuming that symbionts have spread in response to selective pressures, two alternatives can be envisaged, depending on whether each of the major secondary symbionts engages in a mutualist interaction with its host or manipulates its reproduction [2,4].

First, mutualistic endosymbionts provide their hosts with higher benefits than costs and are expected to invade the population rapidly [5,20]. The net benefit for a host to house a given endosymbiont might greatly depend on its physiological or ecological requirements. These needs might differ from one host species to the other, notably when these ones interact in different ways with the same environment [11,64]. In the oak weevil communities, the marked ecological differences observed between the four species [37,58] would coincide with unequal probability for a given symbiont to spread. Conversely, we cannot rule out that the marked ecological differences between the four weevil sibling species might be due to the major symbionts having a distinct impact on their host. Hence, the different symbionts might facilitate the partitioning of ecological niches between their hosts by providing them with expanded or new skills, thereby possibly leading to unequal nutritive assistance [6,7,68-71], dispersal capacity [21-24], or ability to resist natural enemies among host species [17-20,72].

Second, the probability for reproductive parasites to successfully invade their host heavily relies on the efficiency of their transmission from mother to offspring. In each of the four weevil species, males and females were frequently and equally infected, which makes male-killing or feminization processes unlikely. For instance, the infection patterns observed would rather be compatible with cytoplasmic incompatibility (CI) [25-27]. Notably, the two strains of *Wolbachia* identified on the basis of *16 s rRNA*, that are found to be the major secondary symbionts of *C. pellitus* and *C. glandium*, show almost complete mutual exclusion at the individual host level (Figure 3), suggesting mutual bidirectional CI. Should this mechanism actually occur, these *Wolbachia* strains would have been prevented from invading *C. elephas* and *C. venosus* populations because of their too low infection rate (reviewed in [73]). Interestingly, endosymbiont-mediated CI has theoretically been shown to facilitate and reinforce reproductive isolation and speciation [25], and such endosymbiont-mediated species enrichment would therefore be expected particularly in communities composed of sibling host species. However, empirical evidence for this mechanism is still scarce and much debated [73-77].

## Conclusions

Our findings raise the question of whether intracellular micro-organisms might contribute to the structure of natural communities of host species competing with each other, either by accelerating their ecological divergence or by reinforcing their reproductive isolation, possibly leading to speciation. Exploring this proposal calls for both theoretical development and empirical investigations describing the endosymbiotic communities housed by various arthropod communities composed of sibling and competing host species. It should then be possible to test the predictions that communities harboring diverse secondary symbionts have greater species richness and include more closely related species than those deprived of symbionts. It would be worth running experiments in parallel to elucidate the actual impact endosymbionts have on their host, which still remains largely unknown in most natural systems.

### Data accessibility

DNA sequences have been deposited at Genbank with the accession numbers AB514497-AB514504, AB507714-AB507716, and JN100059-JN10090. Phylogenetic trees have been deposited at TreeBase (http://purl.org/phylo/treebase/phylows/study/TB2:S13815).

## Competing interests

The authors declare that they have no competing interests.

## Authors’ contributions

This study is part of the PhD thesis of AM who is focused on the implication of endosymbionts on the structure of sibling insect communities. MCBV and AH supervised this PhD at the interface between community ecology and endosymbiosis interactions. MCBV and SV suggested the exploration of endosymbiotic communities within competing species, supervised and contributed to the ecological research. FM provided his experience about weevil ecology and contributed to the experimental scheduling. AH and FV gave their expertise in the interactions existing between insects and their bacterial endosymbionts. AV conducted the fine dissections of the female ovaries and molecular analyses from DNA purification, cloning, up to sequencing. HH contributed to the methodological setting up of diagnostic PCRs and supervised the phylogenetic analyses. All authors read and approved the final manuscript.

## Authors’ information

MCBV, SV and FM are focused on evolutionary and community ecology. FV, AH, HH and AV have interest in associations between insects and bacteria endosymbionts. This work is part of the PhD of AM who develops an integrative research project linking ecological and symbiotic aspects.

## Supplementary Material

Additional file 1 **Identification of the clones sequenced in the four weevil species.** To identify the endosymbiotic diversity, clones were sequenced in adult females belonging to the four weevil species and live-trapped in two sites (see Methods). We described the best hit blast of each clone and the ratio of the number of similar nucleotides to the total number of nucleotides (called Identities). Click here for file

## References

[B1] Feldhaar H Gross R Immune reactions of insects on bacterial pathogens and mutualists Microbes Infect 2008 10 1082 1088 10.1016/j.micinf.2008.07.010 18672091

[B2] Clark EL Karley AJ Hubbard SF Insect endosymbionts: manipulators of insect herbivore trophic interactions? Protoplasma 2010 244 25 51 10.1007/s00709-010-0156-2 20495935

[B3] Ferrari J Vavre F Bacterial symbionts in insects or the story of communities affecting communities Phil Trans Biol Sci 2011 366 1389 1400 10.1098/rstb.2010.0226 PMC3081568 21444313

[B4] Moran NA McCutcheon JP Nakabachi A Genomics and evolution of heritable bacterial symbionts Annu Rev Genet 2008 42 165 190 10.1146/annurev.genet.41.110306.130119 18983256

[B5] Himler AG Adachi-Hagimori T Bergen JE Kozuch A Kelly SE Tabashnik BE Chiel E Duckworth VE Dennehy TJ Zchori-Fein E Hunter MS Rapid spread of a bacterial symbiont in an invasive whitefly is driven by fitness benefits and female bias Science 2011 332 254 256 10.1126/science.1199410 21474763

[B6] Douglas AE Nutritional interactions in insect-microbial symbioses: aphids and their symbiotic bacteria *Buchnera* Annu Rev Entomol 1998 43 17 37 10.1146/annurev.ento.43.1.17 15012383

[B7] McCutcheon JP McDonald BR Moran NA Convergent evolution of metabolic roles in bacterial co-symbionts of insects Proc Natl Acad Sci U S A 2009 106 15394 15399 10.1073/pnas.0906424106 19706397 PMC2741262

[B8] Lamelas A Gosalbes MJ Moya A Latorre A New clues about the evolutionary history of metabolic losses in bacterial endosymbionts, provided by the genome of *buchnera aphidicola* from the aphid *cinara tujafilina* Appl Environ Microbiol 2011 77 4446 4454 10.1128/AEM.00141-11 21571878 PMC3127723

[B9] Leonardo TE Muiru GT Facultative symbionts are associated with host plant specialization in pea aphid populations Proc R Soc B 2003 270 S209 S212 10.1098/rsbl.2003.0064 14667385 PMC1809968

[B10] Simon J-C Carré S Boutin M Prunier-Leterme N Sabater-Mun B Latorre A Bournoville R Host-based divergence in populations of the pea aphid: insights from nuclear markers and the prevalence of facultative symbionts Proc R Soc B 2003 270 1703 1712 10.1098/rspb.2003.2430 12964998 PMC1691435

[B11] Tsuchida T Koga R Fukatsu T Host plant specialization governed by facultative symbiont Science 1989 2004 303 10.1126/science.1094611 15044797

[B12] Gottlieb Y Ghanim M Chiel E Gerling D Portnoy V Steinberg S Tzuri G Horowitz AR Belausov E Mozes-daube N Kontsedalov S Gershon M Gal S Katzir N Zchori-fein E Identification and localization of a *rickettsia* sp. In *bemisia tabaci* (homoptera: aleyrodidae) Appl Environ Microbiol 2006 72 3646 3652 10.1128/AEM.72.5.3646-3652.2006 16672513 PMC1472322

[B13] Chiel E Gottlieb Y Zchori-Fein E Mozes-Daube N Katzir N Inbar M Ghanim M Biotype-dependent secondary symbiont communities in sympatric populations of *bemisia tabaci* Bull Entomol Res 2007 97 407 413 10.1017/S0007485307005159 17645822

[B14] Gueguen G Vavre F Gnankine O Peterschmitt M Charif D Chiel E Gottlieb Y Ghanim M Zchori-Fein E Fleury F Endosymbiont metacommunities, mtDNA diversity and the evolution of the *bemisia tabaci* (hemiptera: aleyrodidae) species complex Mol Ecol 2010 19 4365 4378 10.1111/j.1365-294X.2010.04775.x 20723069

[B15] Toju H Fukatsu T Diversity and infection prevalence of endosymbionts in natural populations of the chestnut weevil: relevance of local climate and host plants Mol Ecol 2010 20 853 868 21199036 10.1111/j.1365-294X.2010.04980.x

[B16] Montllor CB Maxmen A Purcell AH Facultative bacterial endosymbionts benefit pea aphids *acyrthosiphon pisum* under heat stress Ecol Entomol 2002 27 189 195 10.1046/j.1365-2311.2002.00393.x

[B17] Oliver KM Russell JA Moran NA Hunter MS Facultative bacterial symbionts in aphids confer resistance to parasitic wasps Proc Natl Acad Sci U S A 2003 100 1803 1807 10.1073/pnas.0335320100 12563031 PMC149914

[B18] Haine ER Moret Y Siva-jothy MT Rolff J Antimicrobial defense and persistent infection in insects Science 2008 322 1257 1259 10.1126/science.1165265 19023083

[B19] Brownlie JC Johnson KN Symbiont-mediated protection in insect hosts Trends Microbiol 2009 17 348 354 10.1016/j.tim.2009.05.005 19660955

[B20] Jaenike J Unckless R Cockburn SN Boelio LM Perlman SJ Adaptation via symbiosis: recent spread of a *Drosophila* defensive symbiont Science 2010 329 212 215 10.1126/science.1188235 20616278

[B21] Grenier A-M Nardon C Nardon P The role of symbiotes in flight activity of *Sitophilus* weevils Entomol Exp Appl 1994 70 201 208 10.1111/j.1570-7458.1994.tb00748.x

[B22] Heddi A Grenier AM Khatchadourian C Charles H Nardon P Four intracellular genomes direct weevil biology: nuclear, mitochondrial, principal endosymbiont, and *Wolbachia* Proc Natl Acad Sci U S A 1999 96 6814 6819 10.1073/pnas.96.12.6814 10359795 PMC21998

[B23] Leonardo TE Mondor EB Symbiont modifies host life-history traits that affect gene flow Proc R Soc B 2006 273 1079 1084 10.1098/rspb.2005.3408 16600884 PMC1560252

[B24] Goodacre SL Martin OY Bonte D Hutchings L Woolley C Ibrahim K Thomas CFG Hewitt GM Microbial modification of host long-distance dispersal capacity BMC Biol 2009 7 32 10.1186/1741-7007-7-32 19545353 PMC2706808

[B25] Bordenstein SR Bourtzis K, Miller TA Symbiosis and the origin of species Insect Symbiosis 2003 Boca Raton, FL: CRC Press 283 304

[B26] Werren JH Baldo L Clark ME *Wolbachia*: master manipulators of invertebrate biology Nat Rev Microbiol 2008 6 741 751 10.1038/nrmicro1969 18794912

[B27] Merçot H Poinsot D Infection by *Wolbachia*: from passengers to residents C R Biol 2009 332 284 297 10.1016/j.crvi.2008.09.010 19281959

[B28] Müller JP Hauzy C Hulot FD Ingredients for protist coexistence: competition, endosymbiosis and a pinch of biochemical interactions J Anim Ecol 2011 81 222 232 21831194 10.1111/j.1365-2656.2011.01894.x

[B29] Patot S Allemand R Fleury F Varaldi J An inherited virus influences the coexistence of parasitoid species through behaviour manipulation Ecol Lett 2012 15 603 610 10.1111/j.1461-0248.2012.01774.x 22487404

[B30] Harnett DC Wilson GWT Mycorrhizae influence plant community structure and diversity in tallgrass prairie Ecology 1999 80 1187 1195 10.1890/0012-9658(1999)080[1187:MIPCSA]2.0.CO;2

[B31] Reynolds HL Packer A Bever JD Clay K Grassroots ecology: plant-microbe-soil interactions as drivers of plant community structure and dynamics Ecology 2003 84 2281 2291 10.1890/02-0298

[B32] Van der Heijden MGA Bardgett RD Straalen MV The unseen majority: soil microbes as drivers of plant diversity and productivity in terrestrial ecosystems Ecol Lett 2006 11 296 310 18047587 10.1111/j.1461-0248.2007.01139.x

[B33] Bever JD Dickie IA Facelli E Facelli JM Klironomos J Moora M Rillig MC Stock WD Tibbett M Zobel M Rooting theories of plant community ecology in microbial interactions Trends Ecol Evol 2010 25 468 478 10.1016/j.tree.2010.05.004 20557974 PMC2921684

[B34] Hoffmann A Faune de France Coléoptères Curculionides (Deuxième partie) 1954 Paris, France: Fédération Française des Sociétés de Sciences Naturelles

[B35] Coutin R Original characteristics of the evolving cycles of some European weevil species: *Curculio elephas Gyll., C. nucum L., C. glandium Marsh., C. venosus Grav.* and *C. villosus F* Memoires de la Societe royale belge d’Entomologie 1992 35 259 266

[B36] Hughes J Vogler AP The phylogeny of acorn weevils (genus *curculio*) from mitochondrial and nuclear DNA sequences: the problem of incomplete data Mol Phylogenet Evol 2004 32 601 615 10.1016/j.ympev.2004.02.007 15223041

[B37] Venner S Pélisson PF Bel-Venner MC Débias F Rajon E Menu F Coexistence of insect species competing for a pulsed resource: toward a unified theory of biodiversity in fluctuating environments Public Library of Science One 2011 6 e18039 21445318 10.1371/journal.pone.0018039 PMC3061935

[B38] Toju H Hosokawa T Koga R Nikoh N Meng XY Kimura N Fukatsu T “*Candidatus* curculioniphilus buchneri,” a novel clade of bacterial endocellular symbionts from weevils of the genus *curculio* Appl Environ Microbiol 2010 76 275 282 10.1128/AEM.02154-09 19880647 PMC2798621

[B39] Schauff ME Collecting and preserving insects and mites: tools and techniques 1986 Washington: USDA Misc Publ n°1443: Museum of Natural History

[B40] Sambrook J Russell DW Molecular cloning: a laboratory manual 2001 3 New York, NY: Cold Spring Harbor Laboratory Press

[B41] Pélisson PF Henri H Bel-Venner MC Allemand R Merville A Menu F Venner S Identification at the larval stage of four *curculio* species coexisting on oak trees using PCR-RFLP Entomol Exp Appl 2011 138 77 82 10.1111/j.1570-7458.2010.01077.x

[B42] Simon C Frati F Beckenbach A Crespi B Liu H Flook P Evolution, weighting, and phylogenetic utility of mitochondrial gene sequences and a compilation of conserved polymeras chain reaction primers Ann Entomol Soc Am 1994 87 651 701

[B43] Lefèvre C Charles H Vallier A Delobel B Farrell B Heddi A Endosymbiont phylogenesis in the dryophthoridae weevils: evidence for bacterial replacement Mol Biol Evol 2004 21 965 973 10.1093/molbev/msh063 14739242

[B44] Werren JH Windsor DM *Wolbachia* infection frequencies in insects: evidence of a global equilibrium? Proc R Soc B 2000 267 1277 1285 10.1098/rspb.2000.1139 10972121 PMC1690679

[B45] Duron O Never completely trust a model: insights from cytoplasmic incompatibility beyond *wolbachia-drosophila* interactions Heredity 2008 101 473 474 10.1038/hdy.2008.113 18941470

[B46] Edgar RC MUSCLE: a multiple sequence alignment method with reduced time and space complexity BMC Bioinforma 2004 5 1 19 10.1186/1471-2105-5-1 PMC517706 15318951

[B47] Posada D jModelTest: phylogenetic model averaging Mol Biol Evol 2008 25 1253 1256 10.1093/molbev/msn083 18397919

[B48] Guindon S Gascuel O A simple, fast, and accurate algorithm to estimate large phylogenies by maximum likelihood Syst Biol 2003 52 696 704 10.1080/10635150390235520 14530136

[B49] Ronquist F Huelsenbeck JP MrBayes 3: Bayesian phylogenetic inference under mixed models Bioinformatics 2003 19 1572 1574 10.1093/bioinformatics/btg180 12912839

[B50] Shimodaira H Hasegawa M Multiple comparisons of loglikelihoods with applications to phylogenetic inference Mol Biol Evol 1999 16 1114 1116 10.1093/oxfordjournals.molbev.a026201

[B51] Shimodaira H An approximately unbiased test of phylogenetic tree selection Syst Biol 2002 51 492 508 10.1080/10635150290069913 12079646

[B52] Shimodaira H Hasegawa M CONSEL: for assessing the confidence of phylogenetic tree selection Bioinformatics 2001 17 1246 1247 10.1093/bioinformatics/17.12.1246 11751242

[B53] Meier-Kolthoff JP Auch AF Huson DH Göker M COPYCAT: cophylogenetic analysis tool Bioinformatics 2007 7 898 900 17267434 10.1093/bioinformatics/btm027

[B54] Legendre P Desdevises Y Bazin E A statistical test for host-parasite coevolution Syst Biol 2002 51 217 234 10.1080/10635150252899734 12028729

[B55] Fukatsu T Shimada M Molecular characterization of *rickettsia* sp in a bruchid beetle *kytorhinus sharpianus* Appl Entomol Zool 1999 34 391 397

[B56] Sork VL Bramble J Sexton O Ecology of mast-fruiting in three species of North American deciduous oaks Ecology 1993 74 528 541 10.2307/1939313

[B57] Leibold MA McPeek MA Coexistence of the niche and neutral perspectives in community ecology Ecology 2006 87 1399 1410 10.1890/0012-9658(2006)87[1399:COTNAN]2.0.CO;2 16869414

[B58] Pélisson PF Bel-Venner MC Rey B Burgevin L Martineau F Fourel F Lecuyer C Menu F Venner S Contrasted breeding strategies in four sympatric sibling insect species: when a proovigenic and capital breeder copes with a stochastic environment Funct Ecol 2012 26 198 206 10.1111/j.1365-2435.2011.01925.x

[B59] Mansour K On the so-called symbiotic relationship between coleopterous insect and intracellular microorganisms Q J Microsc Sci 1934 77 255 272

[B60] Boyle L O’Neill SL Robertson HM Karr TL Interspecific and intraspecific horizontal transfer of *wolbachia* in *drosophila* Science 1993 260 1796 1799 10.1126/science.8511587 8511587

[B61] Sandström JP Russell JA White JP Moran NA Independent origins and horizontal transfer of bacterial symbionts of aphids Mol Ecol 2001 10 217 228 10.1046/j.1365-294X.2001.01189.x 11251800

[B62] Russell JA Latorre A Sabater-Muñoz B Moya A Moran NA Side-stepping secondary symbionts: widespread horizontal transfer across and beyond the Aphidoidea Mol Ecol 2003 12 1061 1075 10.1046/j.1365-294X.2003.01780.x 12753224

[B63] Russell JA Moran NA Horizontal transfer of bacterial symbionts: heritability and fitness effects in a novel aphid host Society 2005 71 7987 7994 10.1128/AEM.71.12.7987-7994.2005 PMC1317397 16332777

[B64] Tsuchida T Koga R Horikawa M Tsunoda T Maoka T Matsumoto S Simon JC Fukatsu T Symbiotic bacterium modifies aphid body color Science 2010 330 1102 1104 10.1126/science.1195463 21097935

[B65] Huigens ME de Almeida RP Boons PAH Luck RF Stouthamer R Natural interspecific and intraspecific horizontal transfer of parthenogenesis–inducing *wolbachia* in *trichogramma* wasps Proc Roy Soc Lond B Biol Sci 2004 271 509 515 10.1098/rspb.2003.2640 PMC1691627 15129961

[B66] Mouton L Dedeine F Henri H Boulétreau M Profizi N Vavre F Virulence, multiple infections and regulation of symbiotic population in the *wolbachia-asobara tabida* symbiosis Genetics 2004 168 181 189 10.1534/genetics.104.026716 15454536 PMC1448097

[B67] Oliver KM Moran NA Hunter MS Costs and benefits of a superinfection of facultative symbionts in aphids Proc R Soc B 2006 273 1273 1280 10.1098/rspb.2005.3436 16720402 PMC1560284

[B68] Dowd PF Insect fungal symbionts: a promising source of detoxifying enzymes J Ind Microbiol Biotechnol 1992 9 149 161

[B69] Tokuda G Watanabe H Hidden cellulases in termites: revision of an old hypothesis Biol Lett 2007 3 336 339 10.1098/rsbl.2007.0073 17374589 PMC2464699

[B70] Brownlie JC Cass BN Riegler M Witsenburg JJ Iturbe-Ormaetxe I McGraw EA O’Neill SL Evidence for metabolic provisioning by a common invertebrate endosymbiont, *wolbachia pipientis*, during periods of nutritional stress PLoS Pathog 2009 5 e1000368 10.1371/journal.ppat.1000368 19343208 PMC2657209

[B71] Carpenter KJ Horak A Keeling PJ Phylogenetic position and morphology of spirotrichosomidae (parabasalia): new evidence from *leptospironympha* of *cryptocercus punctulatus* Protist 2010 161 122 132 10.1016/j.protis.2009.06.003 19664955

[B72] Oliver KM Moran NA Hunter MS Variation in resistance to parasitism in aphids is due to symbionts not host genotype Proc Natl Acad Sci U S A 2005 102 12795 12800 10.1073/pnas.0506131102 16120675 PMC1200300

[B73] Engelstädter J Telschow A Cytoplasmic incompatibility and host population structure Heredity 2009 103 196 207 10.1038/hdy.2009.53 19436325

[B74] Shoemaker DD Katju V Jaenike J *Wolbachia* and the evolution of reproductive isolation between *drosophila recens* and *drosophila subquinaria* Evolution 1999 53 1157 1164 10.2307/2640819 28565520

[B75] Marshall JL The *allonemobius-wolbachia* host-endosymbiont system: evidence for rapid speciation and against reproductive isolation driven by cytoplasmic incompatibility Evolution 2004 58 2409 2425 15612285 10.1111/j.0014-3820.2004.tb00871.x

[B76] Telschow A Hammerstein P Werren JH The effect of *wolbachia* versus genetic incompatibilities on reinforcement and speciation Evolution 2005 59 1607 1619 16329235

[B77] Jaenike J Dyer KA Cornish C Minhas MS Asymmetrical reinforcement and *wolbachia* infection in *drosophila* PLoS Pathog 2006 4 e325 10.1371/journal.pbio.0040325 PMC1592313 17032063

